# Psychosocial, emotional and behavioral problems, quality of life, and mental health care seeking behaviors among children and adolescents in Jordan: a national school-based survey

**DOI:** 10.3389/fpubh.2024.1409158

**Published:** 2024-11-12

**Authors:** Yousef Khader, Sara Abu Khudair, Eizaburo Tanaka, Lara Kufoof, Mohannad Al Nsour, Ashraf Aqel, Mohammad Maayeh, Ahmad Kharabsha

**Affiliations:** ^1^Center of Excellence for Applied Epidemiology, The Eastern Mediterranean Public Health Network (EMPHNET), Amman, Jordan; ^2^Department of Community Medicine, Public Health and Family Medicine, Faculty of Medicine, Jordan University of Science and Technology, Irbid, Jordan; ^3^Health Sciences Unit, Faculty of Social Sciences, Tampere University, Tampere, Finland; ^4^College of Arts and Sciences, The University of Tokyo, Meguro, Tokyo, Japan; ^5^School Health Directorate, Jordan Ministry of Health, Amman, Jordan; ^6^School Environment Department, Jordan Ministry of Health, Amman, Jordan

**Keywords:** adolescent, children, mental health, survey, Jordan

## Abstract

**Objectives:**

This study aimed to estimate the prevalence of psychosocial, emotional, and behavioral problems and their symptoms among children and adolescents in Jordan, assess their quality of life, and examine mental health help-seeking behavior.

**Methods:**

A large-scale school-based national survey was conducted in Jordan among children and adolescents aged 8 to 18 years (grades 3 to 12) from the host and refugee populations, utilizing a multi-stage stratified cluster sampling technique to select a nationally representative sample. Two versions of structured questionnaires were used: proxy parent version for students in grades 3 to 6 (8–11 years) and self-report version for students in grades 7 to 12 (12–18 years). The study questionnaires employed internationally recognized and validated tools in English, which were translated into Arabic.

**Results:**

A total of 8,000 (3,433 (42.9%) boys, 4,567 (57.1%) girls) and (3,593 (44.9%) children, 4,407 (55.1%) adolescents) were included. About 24.5% of children had anxiety symptoms (18.0% of Jordanians, 34.5% of Syrian camp refugees, 33.7% of Syrian urban refugees, and 24.7% of Palestinian camp refugees) and 16.6% of children had major depressive disorder symptoms (11.0% of Jordanians, 25.4% of Syrian camp refugees, 25.0% of Syrian urban refugees, and 14.0% of Palestinian camp refugees). Almost 13.9% of children and 19.7% of adolescents had abnormal levels of total emotional and behavioral difficulties. Nearly 16.5% of children and 35.0% of adolescents had poor overall health-related quality of life. When experiencing a personal or emotional problem, only 28.1% of children’s parents would seek help for their children and 19.7% of adolescents would seek help for themselves.

**Conclusion:**

The study revealed a high prevalence of various mental health issues’ symptoms, particularly among refugees and female adolescents. Intention to seek help is relatively low, suggesting that children and adolescents’ mental health needs are not being widely met. It is crucial to implement integrated and coordinated plans and programs that effectively target multiple factors that impact children and adolescents’ mental health, while also respecting the prevailing cultural context. A key aspect of promoting the mental well-being of children and adolescents in Jordan is the inclusive involvement of refugees and individuals from other nationalities.

## 1 Introduction

Childhood and adolescence are critical periods for physiological and psychological development with numerous challenges that could impact mental health. Young refugees are reported to have poorer mental health than non-refugee youngsters (1–4), which could be due to the challenges they and their families face as a result of conflict and war, loss of home, and resettlement in unfamiliar surroundings, as well as the challenges associated with new living conditions and social circumstances (4).

Jordan is a Middle Eastern country with an estimated population of 11,300,000 individuals in 2022 (5). Jordan’s population is relatively young, with approximately 44% of its residents being 19 years old or younger (6). Also, Jordan has one of the highest percentages of refugees *per capita* globally, mostly young population of refugees, with around 54% of refugees 19 years old or younger (6). The majority of refugees in Jordan originate from Syria as a consequence of the 2011 civil war and they usually reside in either refugee camps or urban areas, attending schools under the Ministry of Education’s jurisdiction. This diverse and young population in Jordan highlights the importance of investing in mental health to promote the well-being and healthy development of the younger generation in Jordan. Also, many mental health problems develop in childhood and adolescence and progress later in adulthood, so interventions at a young age are a key factor in improving mental health later in life (7).

A recent scoping review of 28 studies on adolescent mental health in Jordan, encompassing Jordanian, Syrian refugee, and mixed populations, revealed wide variability in the prevalence of mental health conditions (8). The prevalence of depression ranged significantly, from 7.1 to 73.8%, while anxiety rates ranged from 16.3 to 46.8%. Syrian refugee adolescents exhibited markedly higher post-traumatic stress disorder (PTSD) rates (up to 65.1%) compared to Jordanian non-refugees (16.2%). Emotional and behavioral difficulties were also prevalent, with rates ranging from 11.7 to 52.5%. Despite these estimates, many of the studies had limitations that affect the reliability and generalizability of their findings, including small sample sizes, selection and observer biases, and the use of tools lacking proper validity and reliability. Moreover, existing research predominantly focuses on specific mental health conditions, leaving critical gaps in understanding broader emotional and behavioral problems, health-related quality of life, and help-seeking behaviors among Jordanian children and adolescents in Jordan. This study aimed to address these limitations by providing large-scale estimates of the prevalence of mental health issues and emotional difficulties in children and adolescents in Jordan, while also examining their quality of life and help-seeking behaviors. The findings will offer a more comprehensive perspective on youth mental health and inform future interventions.

## 2 Methods

### 2.1 Study design and sampling

A national school-based survey was conducted among Jordanian children and adolescents as well as those of other nationalities and groups such as Syrian and Palestinian refugees aged between 8 and 12 years during the period December 2022–April 2023. This study utilized a multi-stage stratified cluster sampling technique to select a nationally representative sample. For school selection, the sample aimed to achieve coverage of basic and secondary education in the Ministry of Education (MoE), the private sector, and the United Nations Relief and Works Agency (UNRWA) for Palestine refugee schools in Jordan. The school population was stratified into different explicit strata. The first level of stratification is governing authority (MoE, private, and UNRWA). Within these strata, there is implicit stratification by region (North, South, and Central) and governorate (12 governorates). Within the MoE stratum, the second level of explicit stratification is the refugee context (Regular MoE schools and Syrian second-shift schools). Regular MoE schools and MoE Syrian second-shift schools are further stratified by gender (boys, girls, and mixed). Because of its small size, half of the Zaatari refugee camp schools were selected and gender balanced.

A list of all schools was obtained from the MoE. Schools were ordered according to the number of students. A systematic sample was selected from each stratum. All classes (grades 3–12) in the selected schools were included and each class was defined as a unit for data collection. A systematic sample of 30% of students in each class was selected. The decision to select 30% of students from each class was made to increase the number of schools in the study, enhancing overall representativeness. This approach allowed broader coverage across regions, school types, and governing authorities, effectively capturing the diversity of the Jordanian educational system. Furthermore, stratified sampling at multiple levels—governance authority, region, refugee context, and gender—ensured adequate representation of variations across schools and regions. The students were selected proportional to the size of students in the governing authority/governorate strata (self-weighted sample). Those who are not attending or dropped from school were reached and oversampled, by including students attending nonformal education centers. Data was collected specifically from nonformal education centers located in the northern and central regions of Jordan, where the majority of students are enrolled in the non-formal education program. The sample of non-formal education centers was gender balanced.

### 2.2 Sample size

Assuming the prevalence of the common mental disorder (e.g., depression) in children and adolescents equals 25% with a confidence interval width of 4% and confidence level of 95%, the estimated sample size is 1,801. A design effect of 2 to account for the clustering of the observations and a 70% complete response rate are used to adjust the required sample size. To have adequate power to conduct subgroup analysis, the number of potential participants invited to this survey was increased to 9,000 children and adolescents.

### 2.3 Data collection

Prior to data collection, data collectors received extensive training on various aspects of the data collection process, including ethical considerations, the importance of maintaining confidentiality, and best communication practices with the target population. Trained data collectors who visited the selected schools handed out the questionnaire to the target groups, including students in grades 7 to 12 (age 12–18 years), or asked students in grades 3 to 6 (age 8–11) to give the questionnaires to their proxy respondents. Data collectors provided clear information about the purpose of the study, voluntary participation, confidentiality, and anonymity. A written informed consent for their participation was obtained from the legal guardians. For students in grades 7 to 12, the students were asked to maintain adequate physical spacing when completing the questionnaires to minimize the risk of social desirability and to ensure genuine responses. Special care was taken to convey the survey information in clear and understandable language. Principals were contacted in advance to arrange data collection. Data collectors stayed with students to answer questions and maintain privacy.

### 2.4 Study tools

The choice of the tools was guided by a literature review on the topic and by consultation with a group of experts that included a psychologist, a sociologist, a family physician, and two epidemiologists. The study questionnaires are internationally recognized and validated in English. All standardized questionnaires including the students’ and parents’ versions were used after obtaining the approvals of use from the developer or the ones who own the copyright. The survey included two versions, a proxy parent version for parents of students in grades 3 to 6 and a self-report version for students in grades 7 to 12. For the instruments that have only self-report versions, questions were re-worded by the research team to reflect parents’ responses about their children’s cases.

The selected instruments were translated into the Arabic language using a forward-backward method and culturally adapted, in cases where an Arabic language was unavailable. Two bilingual experts independently performed a forward translation of all questionnaires. Based on a consensus meeting a single preliminary Arabic version was formed. This version was translated back into English independently by two other bilingual experts. An expert committee consisting of two forward translators, one backward translator, two clinical health scientists, and one epidemiologist reviewed the original tools and each translated version, which resulted in a pre-final version of the Arabic tool. Special attention was devoted to the clarity of items to allow for similar cognitive processing by the respondents. The survey was anonymous, and respondents had the right to skip questions or discontinue the survey if questions made them feel uncomfortable.

All selected tools were combined and structured in one questionnaire. The first section of the questionnaire included questions related to schools’ characteristics, socio-demographic characteristics, and health status. *The Family Affluence Scale (FAS)-III* (9), was used to examine the SES of children and adolescents. FAS is a self-administered questionnaire comprising six items that assess the family material assets or activities (e.g., number of cars and dishwashers). The six questions were revised to fit the country context and only slightly changed by just making them more relevant to the conversational language style in Arabic. The response options for the questions varied depending on the specific question being asked and included the following options and scores: No/Not at all/None = 0, Yes/One/Once = 1, Two/twice = 2, and more than two/ more than twice = 3. The Participants’ responses were summed and ranged from 0 to 13, with higher scores indicating higher levels of affluence. The following ranges were used to categorize the results for both boys and girls: 0–7 (low affluence), 8–11 (medium affluence), and 12–13 (high affluence) (10).

*The Revised Child Anxiety and Depression Scale (RCADS)* was used to measure symptoms of anxiety and depression in children and adolescents aged between 8 to 18 years (11). The RCADS consists of 47 items rated on a 4-point Likert scale (i.e., never = 0, sometimes = 1, often = 2, and always = 3). The scale yields two total scales, and 6 subscales, corresponding with the Diagnostic and Statistical Manual of Mental Disorders, Fourth Edition (DSM-IV) classifications for anxiety and depressive disorders. The 6 subscales include Separation Anxiety Disorder (SAD), Generalized Anxiety Disorder (GAD), panic disorder (PD), Social Phobia (SP), Obsessive Compulsive Disorder (OCD), and Major Depressive Disorder (MDD). The two total scales include the Total Anxiety Score (the sum of the five anxiety subscales) and the Total Internalizing Scale (the sum of all six subscales). The RCADS is available in two versions, a self-report and a parent version, both of which capture symptoms of anxiety and depression across all total scales and subscales (11). RCADS scores were calculated using an automated Excel scoring sheet publicly available by the developer (11). The scoring sheet utilized United States Norms and required input of grades and gender. RCADS was scored using U.S. norms because the RCADS is a widely validated tool that has been extensively used in research settings in the United States, and the available scoring sheet utilizes these established norms. Raw scores were first calculated and then converted to T-scores. In the current study, cut-off points of T scores of 70 or more were used to categorize those with anxiety and depression symptoms for all total scales and subscales, following the recommendations stated in the RCADS user guide (11). According to the guide, a converted score of 70 or more indicates a clinical range of symptoms (i.e., scores above the clinical threshold) (11).

*The Strengths and Difficulties Questionnaire (SDQ)* was used to assess emotional and behavioral problems. SDG is a brief emotional and behavioral screening questionnaire for children and young people, asking about 25 attributes, some positive and others negative (12). The SDQ is available in self-report and parent versions. Respondents were asked to rate the statements on a 3-point Likert scale (not true, somewhat true, and certainly true), with a mixture of positive and negatively phrased items. The 25 items are divided into 5 scales of 5 items each, generating scores for emotional symptoms, conduct problems, hyperactivity, peer problems, and prosocial behavior, for each of the subscales, the scores can range from 0 to 10 scale. A total difficulties score is generated from the sum of the four sub-scales of emotional symptoms, conduct problems, hyperactivity, and peer problems (20 items). Some items were reversed scored. Subscale scores were only calculated if at least three of the five items had been completed. The cutoff values for defining the abnormal attributes for parents’ versions were as follows: total difficulties score (17–40), emotional problems (5–10), conduct problems (4–10), hyperactivity score (7–10), peer problems (4–10) and prosocial behavior (0–4). The cutoff values for defining abnormal attributes for students’ versions were used as follows: total difficulties (20–40), emotional problems (7–10), conduct problems (5–10), hyperactivity (7–10), peer problems (6–10) and prosocial behavior (0–4). Higher scores on the difficulties subscales (emotional problems, conduct problems, hyperactivity, and peer relationship problems) indicate greater levels of difficulties, whereas higher scores on the prosocial behavior subscale indicate positive social behavior.

*The Children’s Impact of Event Scale-13 (CRIES-13)* was used to assess symptoms of childhood PTSD. It is a 13-item measure and can be used with children 8 years and older (13, 14). CRIES-13 is a self-report scale; therefore, a proxy parent version was developed by rewording the self-report scale to reflect the parents’ experiences or perceptions of the occurrence of various comments of people who had stressful lives in the case of their children. The scale consists of an overall scale and three subscales with 4 items measuring intrusion, 4 items measuring avoidance, and 5 items measuring arousal. Items are scored on a four-point nonlinear scale: not at all (0), rarely (1), sometimes (3), and often (5). The overall CRIES score is the total sum of all items. A cut-off score of 30 on the total scale was described as effective for screening cases of PTSD (13) and was therefore used to categorize those with PTSD symptoms from those without.

*The Pediatric Quality of Life Inventory (PedsQL)* was used to assess quality of life. It is a 23-item scale measuring the health-related quality of life in children and adolescents (15). It assesses five domains including physical functioning (eight items), emotional functioning (five items), social functioning (five items), and school functioning (five items). It includes core health dimensions delineated by the World Health Organization (WHO), including role (school) functioning. It has three Summary Scores including, Total Scale Score (23 items), Physical Health Summary Score (8 items), and Psychosocial Health Summary Score (15 items). The generic core scales have parallel child self-report and parent proxy-report formats. Items are rated on a 5-point Likert scale from 0 (never) to 4 (almost always), with a higher score indicating a better health-related quality of life. Items are scored in reverse order and then linearly converted to a 0–100 scale as follows: 0 = 100, 1 = 75, 2 = 50, 3 = 25, 4 = 0. Published cutoff scores were used to categorize the poor quality of life across all domains, including total, physical, emotional, social, and school functioning domains. For scores less than 78, 88, 75, 80, and 70 in the respective domains, individuals were classified as having poor quality of life (16). Higher scores indicate a better quality of life. The General Help-Seeking Questionnaire (GHSQ) is a two-item questionnaire (17) that was used to assess the intention to seek help from a variety of sources and for a variety of problems. The original version contains two questions that capture the intention to seek help and sources of help for general personal and emotional problems and suicidal ideation. Respondents are asked to rate the likelihood of seeking help from formal or informal help sources, on a 7-point Likert scale ranging from 1 = very likely to 7 = very unlikely. GHSQ uses a matrix format that can be modified according to the characteristics of the sample and the needs of the study. In this study, the GHSQ was revised and changed as follows: First, only the question about personal and emotional problems was included as it relates to the goal of the study, with binary responses (yes, no) to identify the most common sources of help for those who wish to seek help. The sources of help were adjusted to reflect the known sources of help for mental health problems among children, adolescents, and parents in Jordan, based on the recommendation of the study’s expert panel. Because the GHSQ is a self-report questionnaire, the research team developed a proxy version for parents of children by rewording the self-report version to capture their intention to seek help for their children’s problems and sources of help. There is no Arabic version for this scale, so the research team conducted a forward-backward translation for both versions. The modifications on the tool involved language translation and cultural adaptations to ensure that the items were relevant and comprehensible for children and adolescents in Jordan. The modified versions of these tools were also pilot-tested.

*The Barriers to Adolescents Seeking Help Scale—Brief Version (BASH-B)* is an 11 items scale that presents reasons why adolescents might not seek professional help by representing barriers relating to self-sufficiency, embarrassment, time, and money constraints, not wanting family to know, the belief that nothing will help, confidentiality breaches, and coercion (18). In this study, the BASH -B was revised and changed as follows: 9 additional barriers were added to the 11 items to account for the religious and cultural barriers that play a major role in shaping attitudes in the Jordanian context. Answers were changed from Likert score form to binary (yes, no) to establish a clear quantification of each barrier. Furthermore, the scale was modified to focus on reasons for not seeking care rather than barriers. Because the BASH-B is a self-report questionnaire, a proxy version for parents was developed by rewording the self-report version to report parents’ reasons for not seeking mental health help for their children’s problems. There is no Arabic version for this scale, so the research team conducted a forward-backward translation for both versions. The modified versions of these tools were also pilot-tested.

Short questions were used to address diet. The questions were adapted from the Many Rivers Short Food Frequency Questionnaire (MRSSFQ) (19). Four questions were used to explore dietary habits in terms of the frequency of eating fruit and vegetable servings per day. One fruit serving was defined as 1 medium piece or 2 small pieces of fruit and this includes all fresh, dried, frozen, and tinned fruit. One vegetable serving was defined as half a cup of cooked vegetables or 1 cup of salad vegetables. Additionally, junk food consumption frequency was examined using names that are commonly used in Jordan. A fourth question addressed dietary behavior in terms of eating while watching TV, or on mobile phones or tablets.

Short questions on physical activity that have been validated by Prochaska et al. (20) were used. Four questions were used to examine the frequency of weekly engagement in light and vigorous physical activity and the estimated time spent watching TV, mobile phones, or tablets for entertainment purposes.

*The “BEARS” instrument* was used to assess sleep disorders. It is divided into five major sleep domains, providing a comprehensive screen for the major sleep disorders affecting children in the 2 to 18 years old range (21). Each sleep domain has a set of age-appropriate “trigger questions” for use in the clinical interview. B = bedtime problems E = excessive daytime sleepiness A = awakenings during the night R = regularity and duration of sleep S = Snoring. The research team developed an Arabic version for this scale using forward-backward translation for parents and children versions.

*The Problematic Internet Use Questionnaire (PIUQ-6)* was used to assess three key factors in problematic internet use (PIU), including obsession (i.e., obsessive thinking about the internet and mental withdrawal symptoms caused by the lack of internet use), neglect (i.e., neglect of basic needs and everyday activities), and control disorder (i.e., difficulties in controlling internet use) (22). A 5-point Likert scale (“never,” “rarely,” “sometimes,” “often,” and “always/almost always”) is used to evaluate how much the given statements characterized the respondents. Scores range from 6 to 30, with higher scores indicating a higher risk of PIU. Since the PIUQ-SF-6 is a self-report questionnaire, a proxy version for parents was developed by rewording the self-report version to report parents’ perspectives. In the current study, a cut-off score of higher than 15 was used to classify those at risk of problematic Internet use, based on the acceptable sensitivity and specificity analyses of one study (22).

Three questions were asked to assess smoking and tobacco use, including the use of conventional cigarettes, electronic cigarettes, and waterpipe (shisha). For each product, a user was defined as a daily or occasional user of the product (i.e., answering every day or some days).

### 2.5 Pilot testing

A pilot test was conducted to evaluate the content of the surveys and the data collection process. The pilot testing was conducted in two schools in Irbid and Mafraq in northern Jordan on November 7th, 2022. The school in Irbid is a girls’ school for Jordanian nationals and the school in Al Mafraq is located in the Zaatari camp for Syrian refugee boys. A total of 30 students from grades seven to twelve, 15 from each school, were asked to complete the student questionnaire, and 30 children from grades three to six, 15 from each school, were asked to send the questionnaire to their parents. The changes identified in the piloting phase were reflected into the parent and student versions in both languages.

### 2.6 Quality assurance

The main researchers were conducting regular data quality assurance by comparing the data entered with the responses on the paper-based questionnaires, along with setting restrictive data ranges on the Excel sheets. The main researchers observed data collection over the course of the study to ensure adherence to data collection procedures. To ensure systematic quality monitoring, a checklist of key data collection quality indicators was used, including whether students were informed of the survey prior to the start of data collection.

### 2.7 Ethical issues

This survey followed the “Declaration of Helsinki” and other relevant local ethical guidelines for medical and health research involving human subjects. Participants and their caregivers were fully informed by the document explaining the details of the study purpose, methods, ethical considerations, the voluntariness of their participation, and that they could withdraw at any time without explanation. All participants were informed that participation is voluntary and that all data will be kept in a safe, locked cabinet with only limited access by the project team. Similarly, participants were informed that if participation causes any stress or discomfort, they could withdraw from the study without any ensuing penalties and conditions. Written informed consent from the caregiver of the participants and informed assent from the participants were obtained. Ethical approvals were obtained from the Institutional Research Committees at Jordan University of Science and Technology (JUST), and Jordan Ministry of Health (MoH).

### 2.8 Statistical analysis

Data analysis was carried out using IBM SPSS 24 (IBM Corp. Released 2016. IBM SPSS Statistics for Windows, Version 24.0. Armonk, NY: IBM Corp.). Frequencies and percentages were used to describe categorical variables. Internal consistency of the scales and subscales was evaluated using Cronbach’s alpha. Values above 0.7 were usually used to indicate good measure (23). The Cronbach’s alphas of the RCADS were good for all subscales and ranged from 0.68 to 0.82 for the parent-report version and from 0.70 to 0.88 for the self-report version, whereas the Cronbach’s alphas for total anxiety were excellent at 0.92 for the parent-report version and 0.94 for the self-report version. For the subscales of the SDQ, Cronbach’s alphas ranged from 0.37 to 0.71 for the parent-report version and 0.38 to 0.73 for the self-report version, indicating good internal consistency for some scales, such as emotional symptoms and prosocial behavior in both versions (~0.70), while the Cronbach’s alphas for overall difficulties were good at 0.80 for the parent-report version and 0.78 for the self-report version. The other scales and subscales assessed, including CRIES-13, the PedsQl subscales, and the total problematic Internet use scale, all had excellent internal consistency, ranging from 0.81 to 0.88 in the parent-reported version and from 0.82 to 0.88 in the self-reported version. Cronbach’s alphas for the total scale AQ were 0.66 for the parent-report version, and 0.63 for the self-report version, indicating questionable reliability.

## 3 Results

### 3.1 Children’s and adolescents’ characteristics

Of a total of 9,020 children and adolescents, 8,000 (3,433 (42.9%) boys and 4,567 (57.1%) girls) (children aged 8–11 and adolescents aged 12–18 years) responded and completed the study questionnaire. Of those, 3,593 (44.9%) were children and 4,407 (55.1%) were adolescents. Jordanians made up the largest group in the sample (57.8% of children and 66.2% of adolescents), followed by Syrian individuals living outside the refugee camps (24.2% of children and 16.8% of adolescents). Table 1 shows the socio-demographic characteristics and health status of the 8,000 children and adolescents.

**Table 1:** The socio-demographic characteristics and health status of 8,000 children and adolescents living in Jordan.

Variable	Children (8–11 years) *n* = 3,593		Adolescents (12–18 years) *n* = 4,407	
	*n*	%	*n*	%
School classification				
Public school	2,325	64.7	2,811	63.8
Private school	330	9.2	717	16.3
UNRWA school	459	12.8	343	7.8
Non-formal education center	2	0.1	86	2.0
Zaatrai Camp school	477	13.3	450	10.2
Gender				
Male	1,474	41.0	1959	44.5
Female	2,119	59.0	2,448	55.5
Nationality				
Jordanian	2078	57.8	2,917	66.2
Syrian living in a camp	549	15.3	510	11.6
Syrian living outside camps	871	24.2	739	16.8
Palestinian living in a camp	94	2.6	76	1.7
Other	1	0.0	165	3.7
Family Affluence				
Low affluence	1,376	38.3	723	16.4
Medium affluence	1912	53.2	2,446	55.5
High affluence	305	8.5	1,238	28.1
Suffering from any chronic disease	215	6.0	369	8.4
Have special needs	392	10.9	689	15.6
Ever diagnosed with a mental illness or disorder	20	0.6	80	1.8

### 3.2 Anxiety and depression

Approximately one-quarter of the included children (24.5%) and adolescents (27.5%) exhibited anxiety symptoms, while a lower rate of 16.6% in children and 21.9% in adolescents exhibited symptoms of major depressive disorder. Table 2 shows the prevalence rates of depression and anxiety symptoms across all subscales and total scales, by gender. In the children’s group and across all anxiety subscales, the prevalence of obsessive-compulsive disorder symptoms was the highest (36.0%), followed by separation anxiety disorder (29.1%), panic disorder (18.8%), social phobia (12.8%), and generalized anxiety disorder (8.5%). Similarly, the prevalence of obsessive-compulsive disorder symptoms was the highest among adolescents (39.8%), followed by separation anxiety disorder (32.5%), panic disorder (31.6%), generalized anxiety disorder (13.9%), and social phobia (6.6%). In all subscales and total scales, the percentage of adolescents with anxiety or depression symptoms was higher in females than in males, especially in separation anxiety (42.1% vs. 20.2%) and obsessive-compulsive disorder (49.2% vs. 28.0%).

**Table 2:** The prevalence of depression and anxiety symptoms among children and adolescents by gender.

Subscales/Total scales	Children (8–11 years) *n* = 3,593			Adolescents (12–18 years) *n* = 4,407		
	Male *n* = 1,474	Female *n* = 2,119	Total *N* = 3,593	Male *n* = 1959	Female *n* = 2,448	Total *N* = 4,407
	%	%	%	%	%	%
Separation Anxiety Disorder (SAD)	26.6	30.9	29.1	20.2	42.1	32.5
Generalized Anxiety Disorder (GAD)	9.1	8.2	8.5	11.2	16.2	13.9
Panic Disorder (PD)	21.6	16.9	18.8	21.4	39.4	31.6
Social Phobia (SP)	14.4	11.8	12.8	4.2	8.5	6.6
Obsessive-Compulsive Disorder (OCD)	39.8	33.3	36.0	28.0	49.2	39.8
Major Depressive Disorder (MDD)	19.3	14.7	16.6	16.3	26.5	21.9
Total Anxiety	27.4	22.4	24.5	17.6	35.4	27.5

Total Anxiety Scale (sum of SAD, GAD, PD, SP, and OCD).

Table 3 shows the prevalence of anxiety and depression symptoms in children and adolescents according to their nationality. Among children, Syrian nationals, including Syrian urban refugees living outside camps and Syrian camp refugees, had the highest rates of depression and anxiety symptoms across all scales and subscales. Separation anxiety symptoms were most common in Syrian camp refugees (44.1%), generalized anxiety disorder in Syrian urban refugees (12.5%), panic disorder in Syrian camp refugees (26.9%), Social phobia in Syrian urban refugees (15.7%), obsessive-compulsive disorder in Syrian camp refugees (48.4%), major depressive disorder in Syrian camp refugees (25.4%), and total anxiety in Syrian camp refugees (34.5%). Among adolescents, non-Jordanians had the highest rates of depression and anxiety symptoms. Palestinian camp refugees in particular had the highest rates across various scales, including obsessive-compulsive disorder symptoms (47.9%), panic disorder (42.7%), total anxiety (36.6%), major depressive disorder (34.2%), and social phobia (9.7%).

**Table 3:** Anxiety and depression symptoms among children and adolescents according to nationality.

Subscales/Total scale	Children (8–11 years)				Adolescents (12–18 years)				
	Jordanians	Syrian camp refugees	Syrian urban refugees	Palestinian camp refugees	Jordanians	Syrian camp refugees	Syrian urban refugees	Palestinian camp refugees	Others
	%	%	%	%	%	%	%	%	%
Separation Anxiety Disorder	20.1	44.1	42.5	21.3	28.5	44.9	38.3	38.4	36.3
Generalized Anxiety Disorder	6.3	10.5	12.5	11.6	13.6	13.6	14.6	21.7	15.3
Panic Disorder	14.5	26.9	24.1	18.9	28.7	39.7	35.5	42.7	36.3
Social Phobia	11.3	14.0	15.7	14.7	6.5	7.7	5.8	9.7	6.9
Obsessive-Compulsive Disorder	29.2	48.4	43.2	47.3	39.4	38.1	41.6	47.9	40.3
Major Depressive Disorder	11.0	25.4	25.0	14.0	19.7	26.7	25.7	34.2	24.7
Total Anxiety	18.0	34.5	33.7	24.7	25.8	33.0	29.7	36.6	27.6

### 3.3 Emotional and behavioral problems

The total difficulties score was abnormally high in 13.9% of children and 19.7% of adolescents. Table 4 summarizes the prevalence of emotional and behavioral problems among children and adolescents by gender. The largest percentage of children had abnormal levels of emotional problems (17.9%), conduct problems (16.1%), peer problems (16.0%), and to a lesser extent, hyperactivity (10.0%) and prosocial behavior (4.2%). The largest percentage of adolescents had abnormal levels of conduct problems (17.6%), peer problems (16.3%), emotional problems (14.1%), and to a lesser extent, hyperactivity (12.3%), and prosocial behavior (11.2%).

**Table 4:** The emotional and behavioral problems among children and adolescents by gender.

Subscales/Scale	Children (8–11 years) *n* = 3,573			Adolescents (12–18 years) *n* = 4,400		
	Male *n* = 1,466	Female *n* = 2,113	Total *N* = 3,573	Male *n* = 1954	Female *n* = 2,446	Total *N* = 4,400
	%	%	%	%	%	%
Emotional symptoms	18.1	17.8	17.9	12.3	15.6	14.1
Conduct problems	22.0	12.0	16.1	19.0	16.5	17.6
Hyperactivity	13.6	7.4	10.0	10.6	13.6	12.3
Peer problems	17.9	14.8	16.0	16.3	16.2	16.3
Abnormal prosocial behavior	5.3	3.5	4.2	13.1	9.7	11.2
Total difficulties	17.5	11.4	13.9	18.3	20.8	19.7

The total difficulties scale score is generated by summing scores from all the subscales except the prosocial scale. Prosocial behavior is a positive trait.

Table 5 shows the prevalence of behavioral and emotional symptoms in children and adolescents by nationality. Syrian children and adolescents living in a camp had higher abnormal overall difficulties than Jordanians, Syrian urban refugees, and Palestinian camp refugees, at 19.3 and 22.2%, respectively. Among children, Syrian camp refugees were the group with the highest rates of emotional problems (25.7%), conduct problems (18.0%), hyperactivity (12.7%), and abnormal prosocial behavior (5.3%). However, the peer problem rate was highest among Palestinian refugees (20.4%). Among adolescents, Syrian camp refugees displayed the highest rates of conduct problems (22.2%) and peer problems (19.3%). Notably, Jordanians had the highest rates of emotional problems (14.3%) and hyperactivity (12.7%). Additionally, Syrian urban refugees had the highest levels of abnormal prosocial behavior (12.7%).

**Table 5:** Behavioral and emotional symptoms among children and adolescents according to nationality.

Subscale/scale	Children (8–11 years)				Adolescents (12–18 years)				
	Jordanians	Syrian camp refugees	Syrian urban refugees	Palestinian camp refugees	Jordanians	Syrian camp refugees	Syrian urban refugees	Palestinian camp refugees	Others
	%	%	%	%	%	%	%	%	%
Emotional problems	14.5	25.7	21.5	16.1	14.3	13.9	13.1	6.6	20.0
Conduct problems	16.4	18.0	14.3	16.1	17.5	22.2	15.0	17.1	16.4
Hyperactivity	10.2	12.7	8.3	4.3	12.7	9.8	11.5	10.5	15.8
Peer problems	14.2	18.8	18.3	20.4	15.7	19.3	15.6	18.4	18.8
Prosocial behavior	4.3	5.3	3.3	4.3	10.7	12.4	12.7	7.9	9.7
Total difficulties	12.5	19.3	13.9	12.9	19.5	22.2	17.8	18.4	24.8

### 3.4 Post-traumatic stress disorder symptoms

The prevalence of PTSD was 24.6% among children (26.0% among males and 23.6% among females) and 35.5% among adolescents (31.1% among males and 38.9% among females). PTSD symptoms were more common in non-Jordanian children and adolescents. Among children, PTSD symptoms were more common among Syrians living outside camps (30.2%) and Syrian camp refugees (27.4%) than among Jordanian children (21.5%) and Palestinian refugee children (20.0%). Among adolescents, PTSD symptoms were more common among Palestinian camp refugees (37.7%), followed by Syrian camp refugees (36.4%), Jordanian children (36.4%), and Syrian urban refugee adolescents (33.8%).

### 3.5 Sleeping problems

Among children, the most common sleeping problem was excessive daytime sleep (36.4%). Among adolescents, most sleeping problems were critically high, specifically excessive daytime sleepiness, and nighttime awakenings, which were reported in about half of adolescents. Among different nationalities of children, problems falling asleep and night awakenings were most common among Syrian urban refugees, 17.0 and 19.7%, respectively. Snoring was more common among Palestinian camp refugees (14.4%). However, excessive daytime sleepiness was prevalent in about one-third of all nationalities, with rates ranging from 32.6 to 37.5%. Among different nationalities of adolescents, all sleeping problems were of relatively similar rates while being critically high for excessive daytime sleepiness, and nighttime awakening. Table 6 shows the percentages of sleeping problems among children and adolescents by their nationality group.

**Table 6:** Sleeping problems among children and adolescents according to nationality.

Variable	Children (8–11 years)				Adolescents (12–18 years)				
	Jordanians	Syrian camp refugees	Syrian urban refugees	Palestinian camp refugees	Jordanians	Syrian camp refugees	Syrian urban refugees	Palestinian camp refugees	Others
	%	%	%	%	%	%	%	%	%
Bedtime problems	11.3	12.8	17.0	10.9	32.2	34.2	35.2	25.4	33.5
Excessive daytime sleepiness	36.2	37.5	36.7	32.6	52.8	46.7	48.9	58.0	53.2
Awakenings during night	14.6	16.7	19.7	18.7	58.3	58.9	58.4	52.8	61.5
Snoring	11.1	11.2	13.6	14.4	17.3	18.0	18.1	13.9	18.2

During school days, a small percentage of children were sleeping insufficiently for less than 7 h (12.2%), compared to about one-third of adolescents who were sleeping insufficiently for less than 7 h (36.9%) during school days. On weekend days, however, the percentage of those who sleep insufficiently for less than 7 h was lower than on school days, at 11.4% among children and 21.5% among adolescents.

### 3.6 Problematic internet use

The percentage of children and adolescents at risk of problematic internet use was 34.8% (37.9% for males and 32.7% for females) and 45.9% (46.2% for males and 45.6% for females), respectively. The percentage of children at risk for problematic Internet use was higher among Jordanians (38.4%), Syrian urban refugees (34.8%), and Palestinian camp refugees (38.5%) compared with Syrian camp refugees (19.9%). Among adolescents, 46.0% of Jordanians, 42.3% of Syrian camp refugees, 46.2% of Syrian urban refugees, and 50.0% of Palestinian camp refugees were at risk of problematic Internet use.

### 3.7 Tobacco use

In adolescents, waterpipe was the most frequently used tobacco product (19.2%), followed by electronic cigarettes (15.1%), and conventional cigarettes (9.8%). The prevalence of smoking in children and adolescents by their nationality group is shown in Table 7. Waterpipe smoking was most prevalent among adolescents of other nationalities (29.0%), followed by comparatively similar percentages among Syrian camp refugees (19.5%), Jordanians (19.0%), and Syrian urban refugees (18.8%), compared with 6.8% among Palestinian camp refugees. Electronic cigarettes were more used by adolescents of other nationalities (18.1%), and to a similar extent by Jordanians, Syrian camp refugees, and Syrian urban refugees, compared with 9.6% among Palestinian camp refugees. Cigarette smoking was most prevalent among Syrian camp refugees (11.9%).

**Table 7:** Smoking percentages according to nationality among children and adolescents.

Smoking product	Children (8–11 years)				Adolescents (12–18 years)				
	Jordanians	Syrian camp refugees	Syrian urban refugees	Palestinian camp refugees	Jordanians	Syrian camp refugees	Syrian urban refugees	Palestinian camp refugees	Others
	%	%	%	%	%	%	%	%	%
Conventional cigarettes	0.2	0.8	0.9	1.2	9.8	11.9	8.7	6.8	10.3
Waterpipe	0.5	1.6	0.9	1.2	19.0	19.5	18.8	6.8	29.0
Electronic cigarettes	0.5	0.2	1.4	0.0	15.3	15.6	14.3	9.6	18.1

### 3.8 Diet and physical activity

Only 15.2 and 16.0% of children and 21.3 and 23.9% of adolescents eat ≥3 servings of fruits and ≥ 3 servings of vegetables per day, respectively. Among children, Syrian refugees in urban areas and camps were the groups with the lowest percentage of children eating ≥3 servings of fruits and vegetables. Among adolescents, the proportion of those eating ≥3 servings of fruit was also the lowest among Syrian refugees in camps and urban areas, whereas the proportion of those eating ≥3 servings of vegetables was lowest among Palestinian refugees in camps.

About one-third of children (30.3%) and adolescents (36.9%) do not engage in any form of light physical activity per week, with a higher proportion among Syrian children in urban areas (37.4%) and adolescents from Palestinian refugee camps (44.9%). Also, about one-half of children (57.1%) and adolescents (46.3%) do not engage in any form of vigorous physical activity per week, with higher proportions among Syrian children in urban areas (62.0%) and adolescents from Palestinian camps (50.7%), as shown in Table 8.

**Table 8:** Diet and Physical activity among children and adolescents according to nationality.

Variable	Children (8–11 years)				Adolescents (12–18 years)				
	Jordanians	Syrian camp refugees	Syrian urban refugees	Palestinian camp refugees	Jordanians	Syrian camp refugees	Syrian urban refugees	Palestinian camp refugees	Others
	%	%	%	%	%	%	%	%	%
Fruits intake (serving/day)									
0	4.2	14.3	12.8	3.3	11.8	12.6	14.0	9.9	15.3
1–2	77.9	74.0	77.7	70.7	66.4	69.0	64.7	67.6	61.8
≥3	18.0	11.7	9.5	26.1	21.7	18.4	21.3	22.5	22.9
Vegetable intake (serving/day)									
0	4.1	7.7	5.9	2.2	11.5	9.5	11.6	12.5	10.3
1–2	78.7	75.8	82.2	76.3	65.0	66.5	63.4	69.4	61.3
≥3	17.2	16.5	12.0	21.5	23.5	24.1	25.0	18.1	28.4
Light activity									
No activity	28.4	26.1	37.4	26.5	37.9	31.4	34.4	44.9	41.7
1–4 days	63.8	65.1	56.8	69.9	54.1	62.1	57.2	49.3	50.4
5+ days	7.8	8.8	5.8	3.6	8.0	6.5	8.5	5.8	7.9
Vigorous Activity									
No activity	54.8	57.9	62.0	59.8	47.6	41.7	44.5	50.7	43.2
1–4 days	41.4	38.7	35.3	35.6	47.0	54.1*	50.1	46.3	52.1
5+ days	3.8	3.4	2.7	4.6	5.4	4.1	5.5	3.0	4.8

### 3.9 Quality of life

The percentages of children and adolescents with poor quality of life are shown in Table 9. Nearly 16.5% of children and 35.0% of adolescents had an overall poor quality of life. Among children, the largest percentage of respondents had poor physical functioning (33.0%). Similarly, among adolescents, the largest percentage of respondents (47.0%) had poor physical functioning. However, the percentages of poor quality of life were generally high across all other domains for adolescents, particularly emotional functioning (44.2%). Among adolescents, females had a higher percentage of poor quality of life in all domains.

**Table 9:** The percentages of poor quality of life domains among children and adolescents by gender.

Domain	Children (8–11 years)			Adolescents (12–18 years)		
	Male	Female	Total	Male	Female	Total
	%	%	%	%	%	%
Poor total quality of life	16.3	16.7	16.5	32.9	36.6	35.0
Poor physical functioning	32.3	33.4	33.0	44.1	49.3	47.0
Poor emotional functioning	18.8	17.4	18.0	41.0	46.6	44.2
Poor social functioning	17.7	17.3	17.5	29.1	30.1	29.6
Poor school functioning	20.5	17.1	18.5	33.3	35.3	34.4

Classified as poor quality of life with scores of less than 78, 88, 75, 80, and 70 for physical, emotional, social, and school functioning, respectively.

Table 10 shows the prevalence of poor quality of life in children and adolescents according to nationality. Among children, Syrians had the highest rates across all domains, with Syrian urban refugees having the highest rates of overall poor quality of life, poor physical functioning, and poor emotional functioning, at 21.2, 42.0, and 25.3%, respectively. Poor social functioning and poor school functioning were the highest among Syrian camp refugees at 22.4 and 24.2%, respectively. Among adolescents, Palestinian refugees had higher rates than other nationalities in some domains, including poor physical functioning (56.6%), poor emotional functioning (55.3%), and social functioning (36.8%).

**Table 10:** Poor quality of life among children and adolescents according to nationality.

Domain	Children (8–11 years)				Adolescents (12–18 years)				
	Jordanians	Syrian camp refugees	Syrian urban refugees	Palestinian camp refugees	Jordanians	Syrian camp refugees	Syrian urban refugees	Palestinian camp refugees	Others
	%	%	%	%	%	%	%	%	%
Total scale	13.6	20.1	21.2	15.8	34.0	34.4	37.1	39.5	41.8
Physical functioning	28.1	36.8	42.0	33.7	45.7	43.3	52.3	56.6	52.4
Emotional functioning	14.2	21.7	25.3	14.7	43.4	42.4	46.7	55.3	45.7
Social functioning	15.8	22.4	18.7	15.8	29.0	28.7	31.0	36.8	35.2
School functioning	15.8	24.2	21.4	17.9	33.9	32.5	37.0	35.5	37.4

### 3.10 Mental health care seeking behavior

In the event of experiencing a personal or emotional problem, approximately one quarter (23.4%) of both parents and adolescents reported that they would seek help, with a higher percentage reported by children’s parents (28.1%) compared to adolescents (19.7%). Table 11 shows mental health help-seeking intentions and sources among children and adolescents. Among parents who would seek help for their children, the majority mentioned they would turn to mental health professionals (80.8%), followed closely by the current or previous spouse (79.3%) then to a lesser extent to a general practitioner or a specialized doctor (73.8%) and school counselor (67.5%). Among adolescents, they would first turn to their parents (81.5%), followed by friends (60.2%), relatives or family members (57.4%), and religious figures (50.6%). However, school counselors and schoolteachers were the least preferred sources of help among adolescents, with only 31.5 and 34.8% of adolescents would seek their help, respectively.

**Table 11:** Mental health help-seeking intention and sources among children and adolescents and associated factors.

Help-seeking intention and sources of help	Children (8–11 years)		Adolescents (12–18 years)	
	*n*	%	*n*	%
Intention to seek help in the event of experiencing a personal or emotional problem	926	28.1	810	19.7
Sources of help				
Parent or spouse	734	79.3	660	81.5
Mental health professional (e.g., psychologist, social worker, counselor)	748	80.8	383	47.3
A general practitioner or a specialized doctor	683	73.8	403	49.8
Other relative or a family member	568	61.3	465	57.4
Religious figure	484	52.3	410	50.6
School counselor	625	67.5	255	31.5
Friend	383	41.4	488	60.2
School teacher	542	58.5	282	34.8

### 3.11 Reasons for not seeking mental health services

In the case of not intending to seek help when children have a personal or emotional problem, the most frequently reported reason by children’s parents was the belief that it is god’s will, and they have to accept fate and destiny as it is (63.9%), the belief that they need to work on their child’s problems (62.9%), parents would solve their child’s psychological problems by themselves (59.9%), and the belief that God will solve the child’ problems at the right time (53.8%). Among adolescents who were unwilling to seek help, the most frequently reported reason was the belief that it is god’s will, and they have to accept fate and destiny as it is (73.73%) and they would solve their psychological problems by themselves (71.3%). Table 12 shows the frequency of a variety of reasons for not seeking mental health services among children and adolescents.

**Table 12:** Reasons for not seeking mental health services among children and adolescents who did not intend to seek help in the event of experiencing a personal or emotional problem.

Reason	Children (8–11 years)		Adolescents (12–18 years)	
	*n*	%	*n*	%
I believe that it is god’s will, and I have to accept fate and destiny as it is	1,515	63.9	2,431	73.7
I would solve my psychological problems by myself	1,420	59.9	2,353	71.3
I think I should work on my own problems	1,491	62.9	2,232	67.7
I believe that God will solve my problems at the right time, there is no need to ask for help	1,275	53.8	2,321	70.4
I like to control every detail of my life	1,082	45.6	2,286	69.3
Adults really cannot understand the problems that I have	1,017	42.9	1753	53.2
No matter what anyone does, this will not change the psychological problems I have	983	41.5	1,631	49.5
I do not like to take things emotionally	878	37.0	1,622	49.2
I do not want my community to know that I have a mental health problem	946	39.9	1,470	44.6
I do not want my family to know that I have a mental health problem	862	36.4	1,349	40.9
My problem would not be kept secret	872	36.8	1,328	40.3
I cannot afford mental health services even if I want to get it	1,029	43.4	1,163	35.3
I do not know where to find or how to get mental health services	766	32.3	1,193	36.2
I do not always have the necessary transportation around that allows access to mental health service sites	885	37.3	1,052	31.9
I cannot afford the transportation costs necessary to reach the mental health services sites	937	39.5	1,018	30.9
I do not have enough time to seek mental health services	629	26.5	1,174	35.6
I am worried about the providers’ attitude toward me	701	29.6	1,011	30.7
I feel too embarrassed to get professional help	550	23.2	1,042	31.6
People might perceive me as crazy	655	27.6	936	28.4
I do not trust mental health care providers	414	17.5	1,042	31.6

## 4 Discussion

This study presents the main findings of a large-scale representative national survey aimed at assessing the prevalence rates of psychosocial, emotional, and behavioral problems and their symptoms among children and adolescents in Jordan. The results show a relatively high prevalence of mental health problems and symptoms among the included adolescents compared to children, and among female adolescents compared to their male counterparts. In children, rates of symptoms were generally higher among non-Jordanians, particularly Syrians, while rates were comparatively similar among adolescents of all nationalities, with particularly high rates among Syrians and Palestinians in some cases.

Understanding the underlying causes of mental health outcomes in our target population requires a multifaceted approach that considers cultural, economic, and social mechanisms. Culturally, the stigma associated with mental illness often prevents individuals from seeking help. Economically, individuals facing financial instability may experience heightened stress, exacerbating mental health issues. Socially, the presence or absence of strong support networks plays a critical role; individuals with robust social ties tend to report better mental health outcomes, as supportive relationships can provide emotional comfort and practical assistance during times of crisis.

### 4.1 Anxiety and depression

The RCADS was used to assess anxiety and depression as a screening tool, which has been reported to be a reliable tool for assessing symptoms of anxiety and depression in children and adolescents in different assessment settings, countries, and languages (24). Depression disorders’ symptom rates ranged from 8.5 to 36.0% in children and from 6.6 to 39.8% in adolescents. Anxiety symptoms were exhibited in about a quarter of children and adolescents.

Previous studies in Jordan reported similarly high rates ranging from 16.3 to 46.8% for anxiety (25–27) and between 7.1 to 73.8% for depression (25–30). However, the reported prevalence rates in previous studies varied significantly due to the use of different assessment tools for screening anxiety and depression, limited representativeness of the samples, and differences in the populations studied. Thus, comparing estimates between previous studies can be difficult. Compared to the results of the “Youth Wellbeing Prevalence Survey 2020,” that used RCADS and the same clinical threshold, the overall prevalence estimates were lower among children and adolescents aged 2 to 19 years living in Northern Ireland, and the ranking of the most common disorders’ symptoms also varied (31). Different rates could be due to micro-level factors, including differences in parents and adolescents’ attitudes toward mental health, and macro-level factors, including the availability and accessibility of mental health services.

Female adolescents showed higher rates of anxiety and depression symptoms compared to males in adolescents, reflecting a similar trend in the majority of subscales in the adolescent population specifically in Northern Ireland (32). Females having higher rates than males can be explained by different factors, all of which are interrelated and could be shared with other countries or be only specific to Jordan. At the societal level, extreme practices of gender discrimination and inequality, as well as extreme traditional gender roles practiced in some communities, may limit girls’ personal development, autonomy, and self-expression, which may contribute to psychological problems such as anxiety. In addition, puberty is a time of remarkable physical changes when certain gender stereotypes and beauty perceptions can be imposed on girls, particularly through the impact that processed photos on social media platforms can have on them. At the policy level, some basic rights of adolescents, especially girls, including sexual and reproductive health information rights, are not afforded in Jordan, leaving them vulnerable to misinformation and uninformed about the physical and cognitive changes that occur during puberty.

Among children, Syrian nationals had the highest rates of anxiety and depression symptoms across all subscales and total scales. These high rates may be attributed to the numerous traumatic and stressful events they have experienced since the beginning of the Syrian civil war, such as displacement, separation from family members, and acceptance into the host society. These rates continue to be high in adolescence for Syrians but in conjunction with higher rates for Palestinian refugees. Adolescence is associated with developmental changes that may lead to higher levels of anxiety, along with increased consciousness. When adolescents live under fear of uncertainty and low socioeconomic status, higher levels of anxiety and depression are to be expected. Although the war in Syria and the influx of refugees are considered more recent compared to the war in Palestine, the consequences appear to be long-lasting, and both groups need to be the subject of further intervention.

### 4.2 Emotional and behavioral problems symptoms

Abnormal emotional and behavioral problems prevalence estimates ranged from 4.2 to 17.9% in children and 11.2 to 17.6% in adolescents. Total difficulties were abnormally high in 13.9% of children and 19.7% of adolescents. The largest percentage of both groups had abnormal emotional, behavioral, and peer problems. When looking at local studies, two previous studies used the SDQ to assess the emotional and behavioral problems among adolescents, with the same cutoff points to define abnormal levels. In the first study, conducted among adolescents in 2018, the prevalence rates of overall difficulties were much higher than in the current study, with 52.5% among Jordanians and 58.2% among Syrians. These higher percentages could be explained by the inclusion of only some governorates; however, it aligns with the current results in showing that peer problems and conduct problems are the most prevalent emotional and behavioral symptoms among adolescents in Jordan (33). The second study reported relatively lower difficulties in Jordanian adolescents and a different ranking of the most common difficulties, possibly due to being conducted in only two schools in Amman (34). Collectively, findings suggest that emotional and behavioral symptoms are prevalent in both Jordanian and Syrian adolescents, particularly concerning peer relationships and conduct problems, before and after the COVID-19 pandemic, taking into account the timing of the studies and the present one.

Peer problems were prevalent in children and adolescents in the current study and it indicates difficulty establishing and maintaining positive relationships with peers, suggesting problems with communication and interpersonal skills. These difficulties in maintaining positive relationships with peers can be attributed to the long periods of COVID-19 lockdown, which had a significant impact on social interactions and social skill development. However, a 2018 study in Jordan similarly reported a high prevalence of peer problems (33), suggesting that this issue remains persistent and should be prioritized in interventions targeting children and adolescents. Conduct problems were also prevalent among both children and adolescents. Children are known to be at greater risk of developing conduct problems if exposed to violence, criminal behavior, maltreatment, strict parenting, or family conflict (35). A previous study also reported the high prevalence of conduct problems in Jordan (33), suggesting the need for more targeted interventions to manage conduct problems. Parenting may play a role in the major difficulties experienced in childhood and adolescence, with one study in Jordan showing that the majority of mothers perceived their parenting style as authoritative (73.5%) (36).

### 4.3 PTSD

PTSD symptoms were exhibited in about a quarter of the children and a third of the adolescents. Elevated PTSD symptoms in children in Syrian refugee camps and urban settings, may be explained by the additional ACEs that Syrian children may face early in life, including registration-dependent access to health care and different lifestyles compared with non-refugee peers. For adolescents, on the other hand, rates are comparatively high, at about one-third across all nationalities, but highest among Palestinian camp refugees. This suggests that interventions should be aimed at both groups, the Syrian refugees and the Palestinians, even if the crisis in Syria is more recent.

### 4.4 Sleeping problems

The prevalence of sleep problems according to the BEARS Sleep Tool was relatively high, especially excessive daytime sleepiness in both children and adolescents and nighttime awakenings in adolescents. The BEAR’S Sleep Tool was used as a quick and “user-friendly” tool that provides comprehensive screening for major sleep disorders in children and adolescents. Similar to the current study findings and using the BEARS instrument, the most common sleep problem among a sample of children in Iran was excessive daytime sleepiness but at a higher level of 62.9% (37). Nighttime awakening and excessive daytime sleepiness were also reported as the most common sleep problems in a Nigerian study using the same instrument among adolescents, but to a lesser extent; 34.6 and 21.0%, respectively (38). Overall, the differences between cultures and countries in sleeping disorders could be explained by late bedtimes linked to sociocultural factors and leisure activities that may occur late at night.

However, studies have shown that the most common cause of daytime sleepiness in children and adolescents is inadequate sleep (39), which may explain the high rates of daytime sleepiness in both groups especially in adolescents. In the current study, about one-third of adolescents sleep insufficiently for less than 7 h (36.9%) more than children (12.2%), while the American Academy of Sleep Medicine recommends that children should regularly sleep 9–12 h per 24 h and adolescents aged 13–18 years should sleep 8–10 h per 24 h (40). Apart from sleep duration, changes in sleep across adolescence are a normal part of development, with sleep architecture changes across adolescence but could be also driven by external factors, particularly school performance-related factors, social issues and communication, and consumption of nicotine, and caffeine (41). Nighttime awakenings were high in more than half of the adolescents and could be explained by the high levels of anxiety symptoms observed in this group when using RCADS, with sleep disturbances, particularly insomnia – highly prevalent in anxiety disorders (42).

### 4.5 Problematic internet use

The percentage of those at risk of problematic internet use was 34.8% in children and 45.9% in adolescents. Problematic Internet use (PIU) is defined as “Internet use that is risky, excessive or impulsive in nature leading to adverse life consequences, specifically physical, emotional, social or functional impairment” (43). Although PIU lacks a standardized definition, it commonly involves concerns about excessive internet use and is sometimes referred to as internet addiction (44). Currently, internet addiction is proposed as a disorder in need of further study for the Diagnostic and Statistical Manual V (DSM-V) (43). In studies focusing on Jordanian adolescents, one study reported severe internet addiction at a lower prevalence of 6.3%, limited to the capital city and public schools (45), and another study reported that about two-thirds (65%) of Jordanian adolescents in northeastern Jordan were severe to moderately addicted (46), with these rates critically different than the current study given the limited sample representativeness and the different tools used.

In general, the high prevalence of PIU in Jordan is not surprising, given the high internet penetration (the percentage of the total population of a given country that uses the internet), which was 88% of the total population in early 2023 (47). In addition, the Internet is used in various aspects of life in Jordan, such as access to services and entertainment. The use of the Internet was further increased during the pandemic COVID-19 and has persisted, including blended learning approaches in education. However, there are additional factors that may have contributed to the high level of PIU. One factor is the tendency of parents to utilize the Internet as a means of providing entertainment for their children, often as a way for them to escape their own problems or frustrations. Meanwhile, teenagers are known to extensively engage with the Internet for activities like gaming and social networking.

### 4.6 Smoking and tobacco use

The current study shows that waterpipe smoking is alarmingly high and is the main tobacco product used by children and adolescents in Jordan, higher among adolescents. When compared to the results of the Global Youth Tobacco Survey, a relatively similar percentage of 18.9% was reported (48). Collectively, waterpipe use among adolescents is a real threat in Jordan especially since it has been illustrated as a gateway for the initiation of smoking in adulthood in Jordan (49). Another issue is that 15.1% of adolescents smoke electronic cigarettes, which is not far from the percentage of waterpipe smoking, suggesting that adolescents are more attracted to flavored products, which are less restricted in Jordan. The widespread use of flavored tobacco products in Jordan can be attributed to several factors. First, flavors enhance the taste and reduce the harshness of tobacco, making these products more appealing, especially to beginners and youth. Waterpipes and electronic cigarettes are also viewed by various age groups in Jordan as part of their social routine and a fashion statement. Moreover, waterpipe products are not heavily regulated in the country. Cafes often lack strict enforcement, and numerous Jordanian companies have recently emerged to supply waterpipes directly to households, leaving regulation largely in the hands of parents and vendors.

Tobacco use is one of the leading preventable causes of death worldwide, disproportionately affecting individuals with mental illness and substance use disorders (50). The mechanisms linking mental health conditions and cigarette smoking are complex and likely differ across each of the various disorders. The prevailing belief is that patients with mental health conditions often smoke to manage or alleviate symptoms associated with their disorder (51). To address this, it is recommended that strict regulations be enforced regarding the availability and provision of waterpipes in restaurants, as well as their home delivery services to adolescents. It is also recommended to regulate the forms of indirect advertising or the use of non-formal platforms such as social media, as well as the provision of all forms of smoking products and their delivery to households. School-based smoking prevention curricula are recommended to focus not only on smoking danger education but also on enhancing social competence and addressing social influences (52).

### 4.7 Diet and physical activity

Consumption of three or more servings of fruit per day was reported by only 15.2% of children and 23.9% of adolescents, and consumption of 3 or more servings of vegetables per day was reported by only 16.0% of children and 21.3% of adolescents. Engaging in no light physical activity per week was reported by 30.3% of children and 36.9% of adolescents, while 57.1% of children and 46.3% of adolescents reported not engaging in vigorous physical activity during the week. Physical inactivity and low intake of fruits and vegetables among children and adolescents in Jordan are widespread and could continue into adulthood and pose a risk for the development of chronic diseases and poorer mental health (53). In the current study, only a very small percentage of children and adolescents consumed 3 or more servings of fruits and vegetables, whereas the WHO recommends at least roughly equivalent to five servings of fruits and vegetables per day (54).

The prevalence of adolescent overweight and obesity is high and increasing in Jordan. These trends are associated with increased adoption of non-healthy lifestyles characterized by unhealthy eating habits such as low intake of fruits, vegetables, and milk, high intake of foods rich in sugar and fat, and inadequate physical activities (55). One review indicated inadequate fruit and vegetable consumption among adolescents in Arab countries in general and reported that the proportion of those who met the recommendations of eating five servings per day ranged between 10 and 29% (56). Studies on fruit and vegetable consumption and physical inactivity are scarce in Jordan and some of the available information are from old studies. Insufficient fruit and vegetable consumption has been previously reported among adolescents aged 13–15 years in Jordan, with a decline observed between the 2004 and 2007 national Global School-based Health Survey (GSHS) data (57–59). Additionally, a 2009 study involving adolescents from both public and private schools in Amman found that the average frequency of vegetable and fruit intake was 4.5 ± 2.1 and 4.2 ± 2.2 times per week, respectively (55).

The low consumption of fruits and vegetables could be explained in several ways. In Jordan, most of the school food particularly in public schools is unhealthy and lacks adequate nutritional value and contains high levels of unhealthy ingredients such as added sugars, unhealthy fats, and processed food (60). Another explanation is that adults in Jordan also consume low fruits and vegetables while the family has a great influence on the intake of fruits and vegetables, as it shapes the eating habits of young people and is the main supplier of food in a household. Unhealthy eating habits put adolescents at risk for chronic diseases and can also lead to nutritional deficiencies that can affect their moods and health-related quality of life.

Children of Syrian refugees in urban areas consumed fewer fruits and vegetables than recommended and were more inactive. This problem may be related to their low socioeconomic status, which limits their ability to purchase fruits and even vegetables. Evidence suggests that diets meeting recommendations for fruit and vegetable intake are more costly and may be a greater constraint on the diet quality of people of lower socioeconomic positions (61). Also, a high proportion of adolescents in the Palestinian refugee camps were physically inactive, which could be related to the proximity of the schools to their homes, as many schools are in the camp area. However, this is also the case among adolescents living in Syrian refugee camps, but only 31.4% did not engage in any form of light physical activity, compared to 44.9% of those living in Palestinian camps. It is possible that UNRWA’s funding deficit (62) and the associated consequences of unmotivated teachers and lack of prioritization led to less attention being paid to physical education or extracurricular activities.

### 4.8 Health related quality of life

Health-related quality of life is a multidimensional concept that encompasses, at the individual level, physical and mental health perceptions (e.g., energy level, mood) and their correlates—including health risks and conditions, functional status, social support, and socioeconomic status (63). In the current study, a considerable percentage of children and adolescents had an overall poor health-related quality of life, particularly among adolescents and in the domain of physical functioning in both age groups, in addition to emotional functioning in adolescents. Having poor physical functioning in childhood and adolescence is expected given the low physical activity levels and unfavorable dietary habits in both age groups and high smoking rates among adolescents reported in the current study. High levels of poor emotional functioning in adolescents may be related to overall increased exposure to adverse childhood experiences, school stressors, and puberty-related changes, along with the high rates of anxiety, depression, and emotional and behavioral problems reported in the current study. When adolescents enter puberty, they often have difficulty coping with their environment and suffer hormonal changes, making them more susceptible to poor emotional functioning.

The reported high poor physical functioning within the Palestinian refugee group can be explained by the findings of the current study, with this group having the highest percentage of not being engaged in light (44.9%) or vigorous (50.7%) physical activities weekly more than other nationalities among adolescents. However, it is important to highlight a broader issue that hinders efforts to address the health-related quality of life, UNRWA is currently undergoing a funding gap that restricts its ability to provide sufficient support to Palestinian refugees (62). Resolving the economic challenges faced by UNRWA is crucial for enhancing the health-related quality of life among Palestinian refugees.

### 4.9 Mental health care seeking behavior

A small percentage of children’s parents or adolescents in Jordan considered seeking help for emotional problems, suggesting that the majority of children and adolescents do not receive the support they need when facing mental health challenges. In other Middle Eastern countries with comparable cultural norms, such as Egypt, help-seeking behavior among adolescents was also notably low. In one study, only 58% of surveyed adolescents in Egypt reported that they would seek help if faced with a mental illness (64). However, in countries like Italy, where mental health is more openly discussed, 91% of adolescents reported a tendency to seek help in case of serious difficulties (65). The low percentage observed in Jordan may be due to a general lack of familiarity with mental health issues, insecurity about seeking help, and a complex intersection of factors that deter help-seeking behavior among children, parents, and adolescents. Help-seeking behavior is a protective factor for young people, important for their mental health, well-being, and development, and needs to be perceived more positively by the community.

When examining sources of help, spouses and parents were the most frequently reported sources of help that will be approached by parents of children and adolescents, respectively, which could be explained by the spiritual and physical closeness and the integral role of the family in Middle Eastern culture. The data also showed that adolescents in the study prefer using informal sources of help after their parents and that they particularly prefer close sources of help and those driven by self-confidence and self-control. Parents, on the other hand, tend to prefer formal sources such as general practitioners or specialized physicians or psychiatrists, and school counselors in addition to their spouses. Such differences in help-seeking sources could provide a basis for more targeted interventions. However, having teachers and counselors as the least preferred sources of help for adolescents suggests that there are gaps in mental health teacher-led interventions and support in Jordan. In Jordan, school-based programs are generally delivered by teachers due to the high cost of hiring mental health professionals. However, the findings of the current study suggest that greater emphasis should be placed on improving the quality of psychosocial support provided by teachers in these programs. Regarding the reasons for not seeking mental health help, a strong belief in self-reliance and self-control was frequently reported, reflecting similar trends observed in other Arab countries (66).

### 4.10 Strengths and limitations

This study has several strengths, including the inclusion of participants from diverse cultural and socio-economic backgrounds, encompassing both local Jordanian youth and refugees. This diversity enhances the generalizability of the findings across various demographic groups. Furthermore, the use of standardized and validated assessment tools for measuring mental health significantly strengthens the research. However, the study also has several limitations. One major concern is underreporting bias, which can significantly affect findings related to mental health conditions such as anxiety, depression, and behavioral problems. Parents may downplay their child’s mental health symptoms due to stigma, a lack of understanding, or the desire to present a positive image of their family, leading to the underreporting of conditions like anxiety and depression. Another potential bias is social desirability bias, where respondents may answer questions in a way they believe will be viewed favorably by others, particularly concerning sensitive topics like mental health.

## 5 Conclusion

The study revealed a high prevalence of various mental health issues and symptoms, with particularly higher rates observed within the refugee community and female adolescents. Intention to seek help is relatively low, suggesting that children and adolescents’ mental health needs are not being widely met. Implementing integrated and coordinated plans that effectively address the multiple factors impacting the mental health of children and adolescents is essential, while also respecting the prevailing cultural context. Key strategies include conducting training programs for teachers and caregivers to recognize and address mental health issues in children and adolescents, integrating mental health services within schools to provide access to counselors trained to work with diverse populations, including refugees, and involving families in the treatment process by offering family therapy options that respect cultural practices and beliefs. Additionally, launching targeted awareness campaigns can educate the public about the importance of mental health and available services, with an emphasis on inclusivity for refugees and marginalized groups. It is also recommended to develop community engagement strategies that involve leaders from various cultural groups in designing mental health programs. Collaborating with local religious leaders can help incorporate culturally appropriate practices into mental health interventions, such as faith-based support groups. Furthermore, addressing potential barriers, such as the stigma surrounding mental health, is crucial. Targeted awareness campaigns should be conducted to educate the community about mental health, emphasizing that seeking help is a sign of strength. Another significant barrier is the lack of access to services; thus, increasing the availability of mental health services in community centers and schools is necessary. Future studies should explore how family structure and dynamics influence mental health outcomes in children and adolescents from refugee backgrounds, assessing both protective and risk factors.

## Data Availability

The raw data supporting the conclusions of this article will be made available by the authors, without undue reservation.
